# Supplementary material for: An artificially-intelligent cornea with tactile sensation enables sensory expansion and interaction
Source: Nat Commun. 2023 Nov 7;14:7181. doi: 10.1038/s41467-023-42240-3 (PMC10630301; doi:10.1038/s41467-023-42240-3)
Supplement: Supplementary file 1 — Supplementary Information [file 41467_2023_42240_MOESM1_ESM.pdf]

Supplementary Information

An artificially-intelligent cornea with tactile sensation enables sensory expansion and interaction

Shangda Qu[1,2,3,4], Lin Sun[1,2,3,4], Song Zhang[1,2,3], Jiaqi Liu[1,2,3], Yue Li[1,2,3], Junchi Liu[1,2,3], and Wentao Xu[1,2,3]*

[1]Institute of Photoelectronic Thin Film Devices and Technology, Key Laboratory of Photoelectronic Thin Film Devices and Technology of Tianjin, College of Electronic Information and Optical Engineering, Engineering Research Center of Thin Film Photoelectronic Technology of Ministry of Education, Nankai University, Tianjin 300350, China

[2]Smart Sensing Interdisciplinary Science Center, Nankai University, Tianjin, 300350, China

[3]Shenzhen Research Institute of Nankai University, Shenzhen 518000, China

[4]These authors contributed equally: Shangda Qu, Lin Sun.

*e-mails: wentao@nankai.edu.cn

Supplementary Methods

1. Fabrication of electrochromic device

The sandwich-type electrochromic device is composed of ITO/PET substrates, electrochromic layer that uses PEDOT: PSS, and gel electrolyte layer that uses $LiClO_4$. Electrochromic ink derived from PEDOT: PSS (CHRO-EP202-P) and electrolyte containing $LiClO_4$ (ENER-EI30M) were purchased from Shanghai Mifang Electronic Technology co. Ltd. The electrochromic device was prepared according to the following steps. First, ITO/PET substrates were treated with UV-ozone for 15 min. The electrochromic ink was spin-coated (1500 rpm, 30 s) on ITO/PET substrate, then annealed at 120 °C for 30 min. The gel electrolyte was spread onto another ITO/PET substrate by using an applicator. Finally, the ITO/PET substrates were assembled to form a sandwich-type electrochromic device.

2. Material characterization and device measurements

The morphology of ZTO fibers was observed using a scanning electron microscope (SEM) (Apreo S, Thermo Scientific) and an optical microscope (OM) (DM2700M, Leica). Atomic force microscopy (AFM) images were collected using a Bruker dimension icon microscope in tapping mode. The crystal features of ZTO fibers were characterized by a high-resolution transmission electron microscope (HRTEM) (JEM-2800, JEOL). X-ray diffraction (XRD) patterns were obtained using a Rigaku Ultima-IV instrument. X-ray photoelectron spectroscopy (XPS) was conducted using an ESCALAB 250Xi instrument (Thermo Scientific). The optical transmission spectra and haze were obtained by UV-Vis-NIR spectrophotometer (Cary 5000, Agilent) and (UV-2450, Shimadzu), respectively. The frequency-dependent capacitance of the ion gel was characterized using a semiconductor parameter analyzer (B1500A, Agilent). All electrical measurements of the artificial synapses were performed in an N_2 environment at room temperature by using a Keithley 4200A semiconductor parameter analyzer and a probe station.

3. First-principles calculations

The 2×2×2 supercells were built using the special quasi-random structure (SQS) approach to obtain the molar ratios of Zn:Sn that were detected in experiments[S1]. The First-principles calculations were performed by the Vienna Ab-initio Simulation Package (VASP), which applies density functional theory (DFT)[S2,3]. According to the generalized gradient approximation (GGA), the projector-augmented wave (PAW) pseudopotentials were used to treat the interaction between the valance electrons and ionic cores[S4], which was parametrized by Perdew-Burke-Ernzerhof (PBE) functions[S5]. The cut-off energy for the wave function was set as 600 eV, and the convergence criterion for self-consistent calculation was set to 10^{-6} eV. The Brillouin zone was sampled by using a Γ-centered mesh with a sampling density of $0.02\times 2\pi$ Å^{-1}. All atoms were fully relaxed until all the residual forces were < 0.02 eV/Å. The amorphous structure was obtained using *ab initio* molecular dynamics (AIMD) simulations with a prototype of SnO_2, where the temperature was fixed at 773 K for 2 ps with an initial time step of 1 fs, and then decreasing to 293 K during 2 ps with a time step of 1 fs. Data postprocessing and band unfolding conducted using Vaspkit code[S6].

4. Construction of artificial synapse-amplifier circuit-electrochromic actuator system

An operational amplifier was used to output the desired voltage to operate the electrochromic actuator. To drive the actuator, for the artificially-intelligent cornea stimulated by mechanical stimuli, the R_A and R_f of the amplifier were set as 2.2 kΩ and 220 kΩ, respectively. For the artificially-intelligent cornea stimulated by light stimuli, R_A and R_f were set as 2.2 kΩ and 100 kΩ, respectively. The Au electrodes of the ZTO artificial synapse were connected to the amplifier circuit to convert currents to output voltages, such that the electrochromic actuator could be operated.

Supplementary Notes

Supplementary Note 1. Calculation of field-effect carrier mobility

The field-effect carrier mobility μ_n was extracted using the following equation:

$$I_{ds} = \frac{WC_i\mu_n}{2L}\left[(V_g - V_{th})V_d - \frac{1}{2}V_d^2\right]^2$$

where W is channel width, L is channel length, C_i is gate capacitance, V_g is gate voltage and V_{th} is threshold voltage. At 1 kHz, the frequency-dependent capacitance of the ion gel is ~ 2.31 μF/cm^{-2} (Supplementary Fig. 12), due to the formation of electrical double layers induced by mobile ions[S7,8]. μ_n [cm^2/(V·s)] was 8.9 for ZTO-7:3, 6.3 for ZTO-1:1, and 12.8 for ZTO-3:7 artificial synapses.

Supplementary Note 2. Calculation of band gap of ZTO fibers

The band gap E_g of ZTO fibers was extracted from the following equation[S9,10]:

$$\alpha h\upsilon = A(h\upsilon - E_g)^n$$

where α is the absorption coefficient, h is Planck's constant, υ is the photon's frequency, A is a constant, E_g is the band gap, and n is 0.5 here.

Supplementary Figures

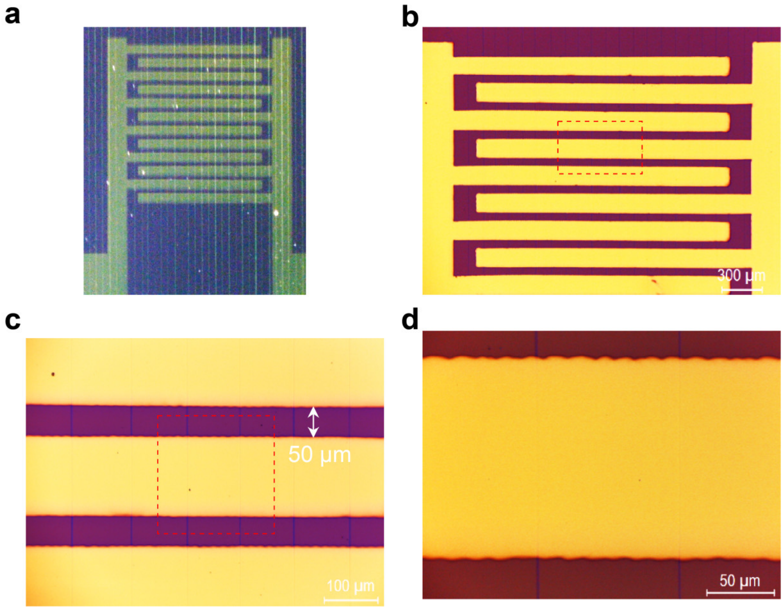

Supplementary Figure 1. Statistics information about number of ZTO fibers and their coverage area of the surface. a Digital image of an artificial synapse. **b, c, d** Optical microscopy images of the device at 5× (**b**), 20× (**c**), and 50× magnification (**d**). **c** corresponds to the area in the dotted box of **b**, and **d** corresponds the dotted box in **c**.

Note: The distance between two adjacent interdigital electrodes is ~ 50 μm. The fibers between two adjacent interdigital electrodes are exposed and covered with ion gel, and the rest are covered with gold electrodes. In each ZTO fiber, 11 segments are exposed to ion gel, so the total length of one fiber covered with ion gel is ~ 550 μm. In addition, ~ 288 Au/ZTO junctions in total engaged in the formation of postsynaptic current.

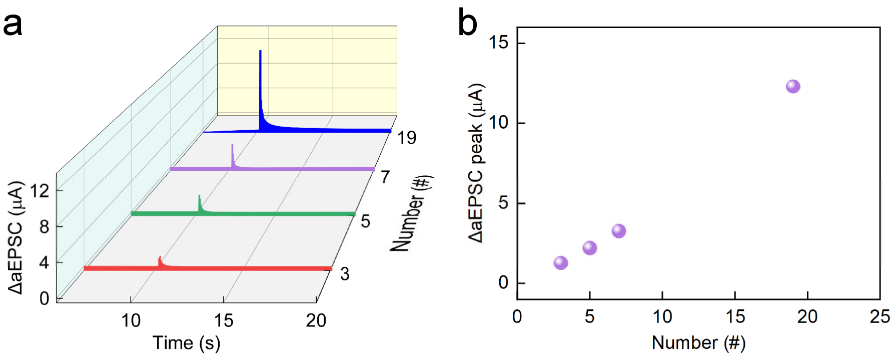

Supplementary Figure 2. a ΔaEPSC of ZTO-3:7 artificial synapse with 3, 5, 7, or 19 ZTO fibers. **b** ΔaEPSC peak of ZTO-3:7 artificial synapse with vs number (3, 5, 7, 19) of ZTO fibers.

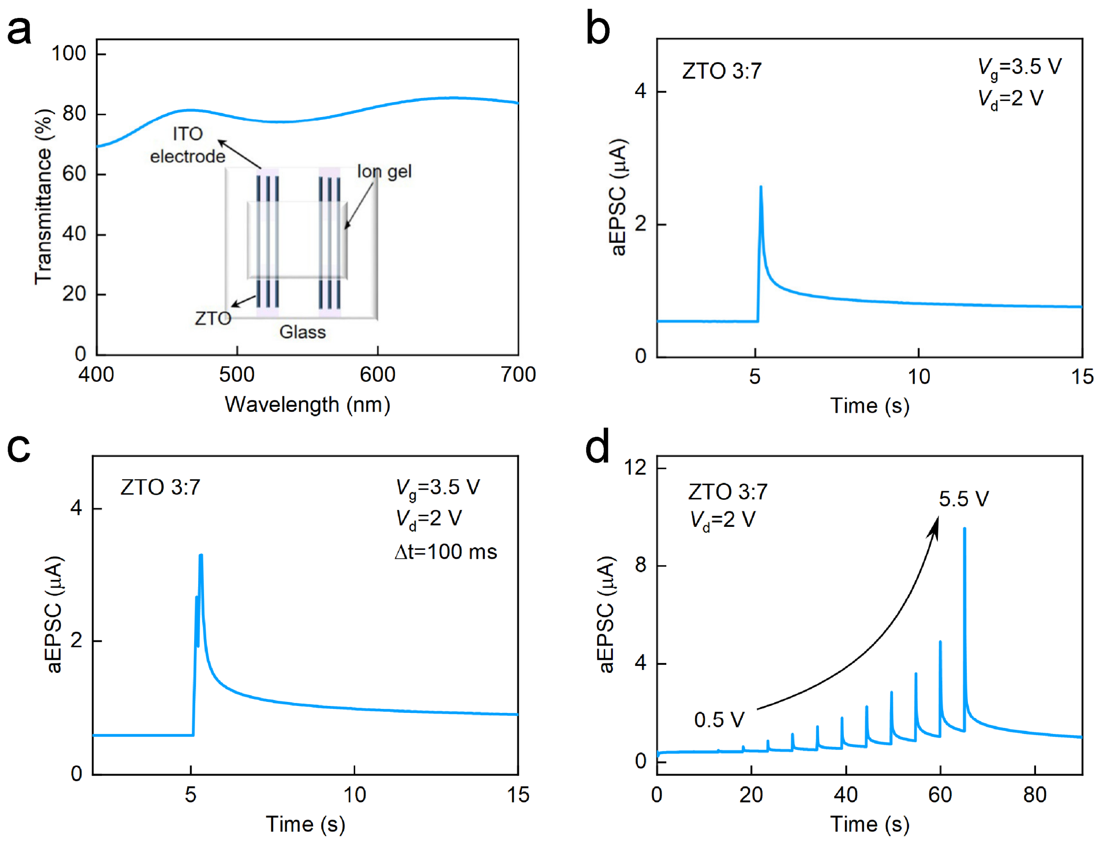

Supplementary Figure 3. Transparency and synaptic plasticity of transparent synaptic device. a Structure and transparency of the entire synaptic device prepared on a glass substrate. **b** aEPSC of the transparent synaptic device triggered by a single spike (3.5 V, 50 ms) at V_d = 2 V. **c** aEPSC of the transparent synaptic device triggered by a pair of spikes (3.5 V, 50 ms) at Δt (time intervals between two spikes) = 100 ms. **d** aEPSC of the transparent synaptic device triggered by spikes with amplitudes 0.5, 1, 1.5, 2, 2.5, 3, 3.5, 4, 4.5, 5, and 5.5 V.

Note: The transparent synaptic device is composed of a glass substrate, patterned ITO electrodes, ZTO-3:7 fibers, and ion gel. The fabrication process of the transparent synaptic device is similar to the ZTO-3:7 artificial synapse prepared on the SiO_2/Si substrate except for the source and drain electrodes. The source and drain electrodes of the transparent synaptic device were patterned ITO obtained by sputtering. The width of ITO electrodes is 3 mm, and the length between source and drain electrodes is 4 mm.

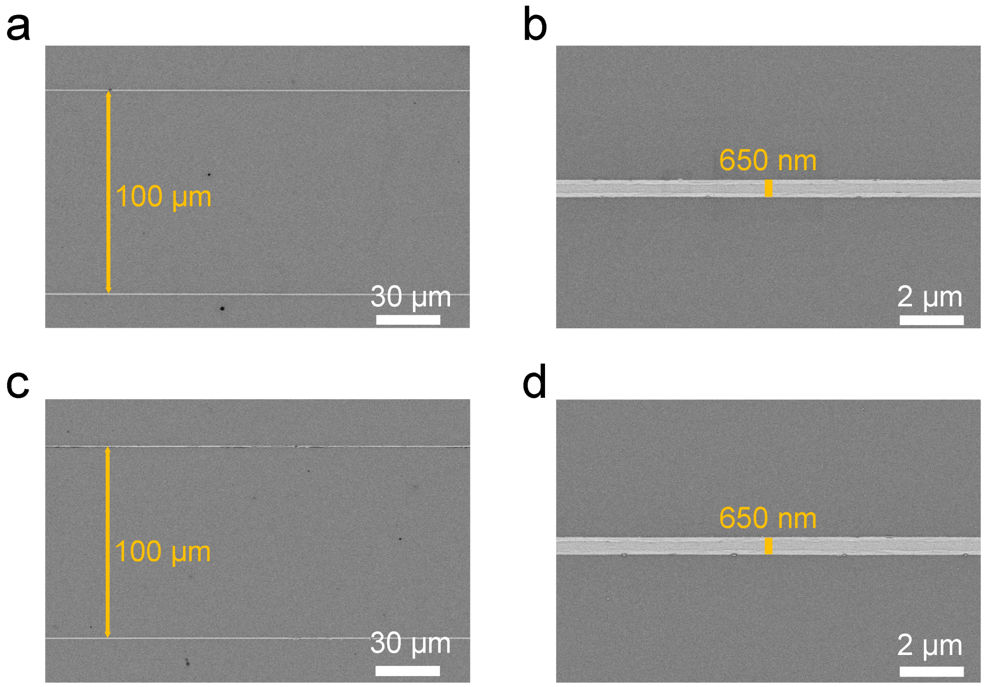

Supplementary Figure 4. SEM images. **a, b** Fibers array and a single fiber of ZTO-7:3. **c, d** Fibers array and a single fiber of ZTO-1:1.

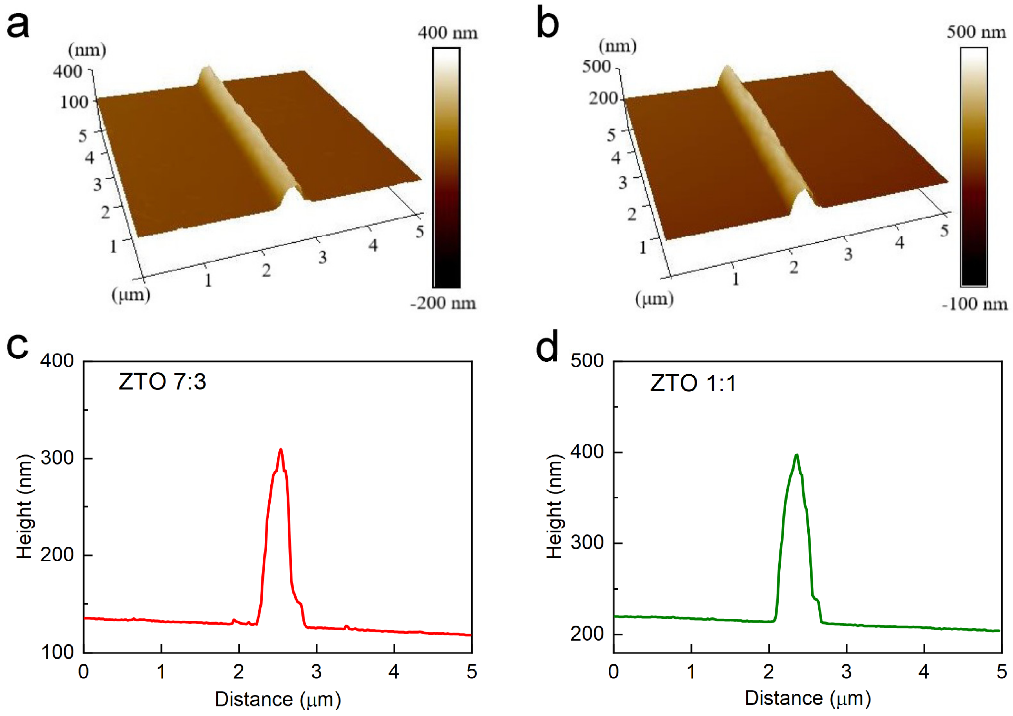

Supplementary Figure 5. AFM images and cross-sectional analysis. **a, c** A single ZTO-7:3 fiber. **b, d** A single ZTO-1:1 fiber.

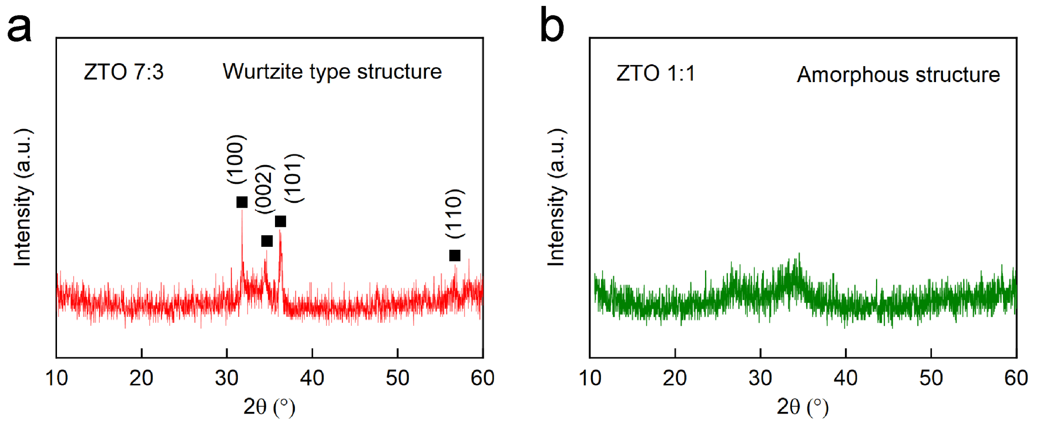

Supplementary Figure 6. XRD patterns. **a** ZTO-7:3 fibers. **b** ZTO-1:1 fibers.

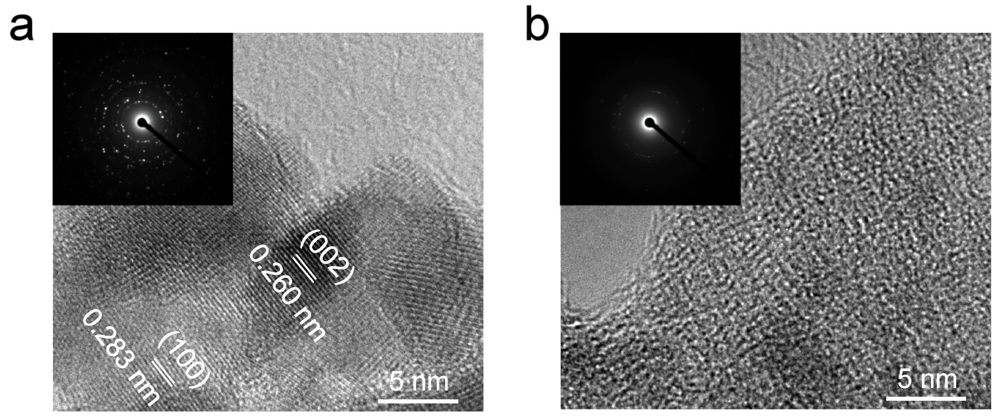

Supplementary Figure 7. High-resolution TEM images. **a** ZTO-7:3 fibers. **b** ZTO-1:1 fibers. Inset: Selected area electron diffraction (SAED) patterns of the corresponding samples.

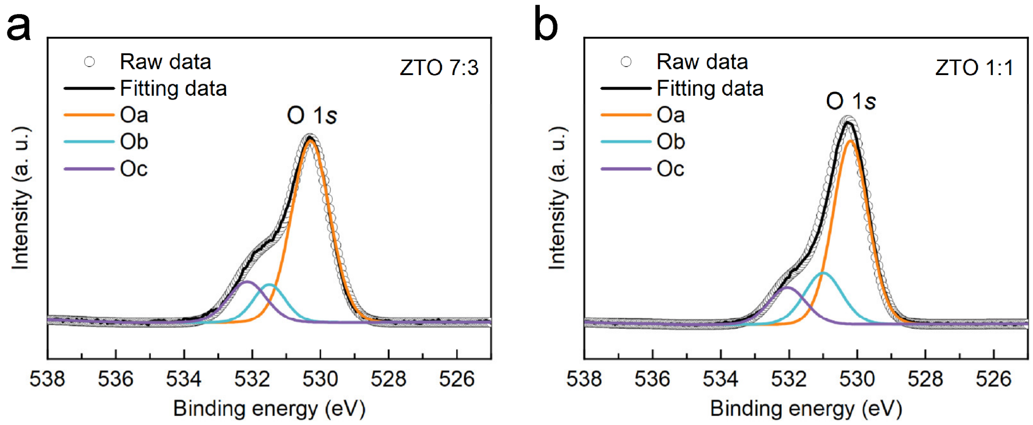

Supplementary Figure 8. O 1*s* XPS spectra. **a** ZTO-7:3 fibers. **b** ZTO-1:1 fibers.

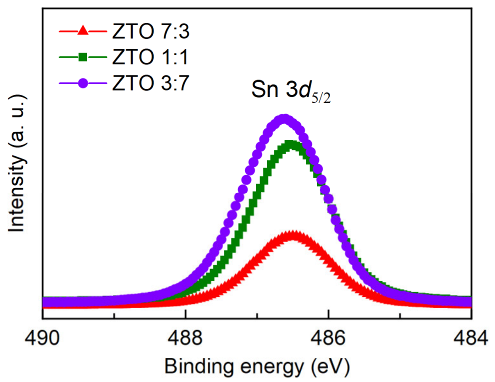

Supplementary Figure 9. Sn $3d_{5/2}$ XPS spectra of the ZTO fibers at various Sn-concentration.

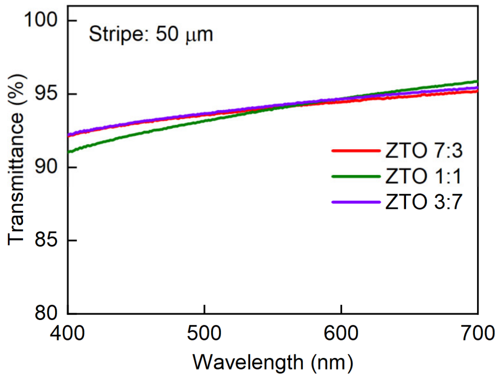

Supplementary Figure 10. Transmittance of different ZTO fibers in visible range.

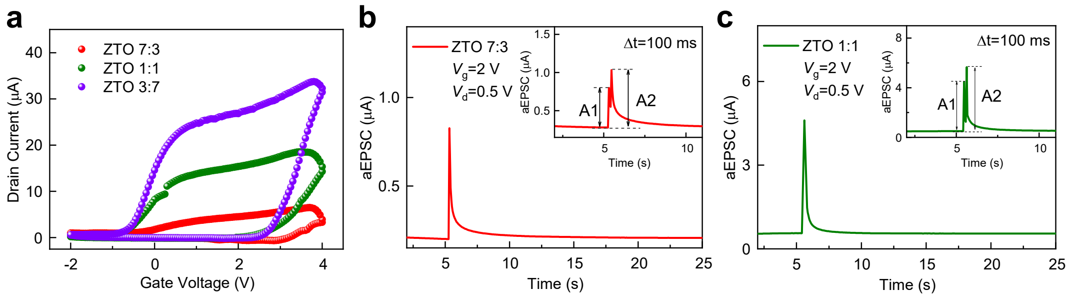

Supplementary Figure 11. Transfer characteristics of ZTO artificial synapses, and aEPSC triggered by a single spike and a pair of spikes. a Transfer characteristics (from -2 to 4 V) of ZTO artificial synapses with different Zn:Sn ratios at drain voltage $V_d = 0.5$ V. **b** aEPSC of ZTO-7:3 artificial synapse triggered by a single spike. Inset: aEPSC triggered by a pair of spikes. **c** aEPSC of ZTO-1:1 artificial synapse triggered by a single spike. Inset: aEPSC triggered by a pair of spikes.

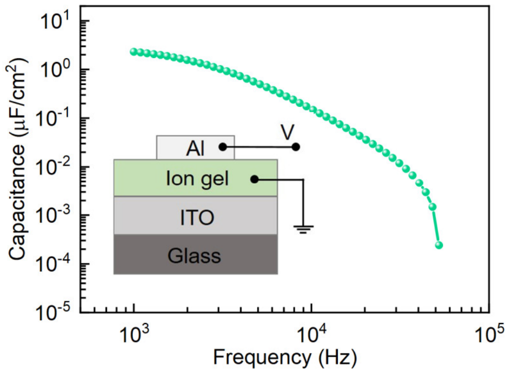

Supplementary Figure 12. Frequency-dependent capacitance of the ion gel. The ion gel was sandwiched between Al and ITO electrodes. Inset: Schematic structure of the test sample.

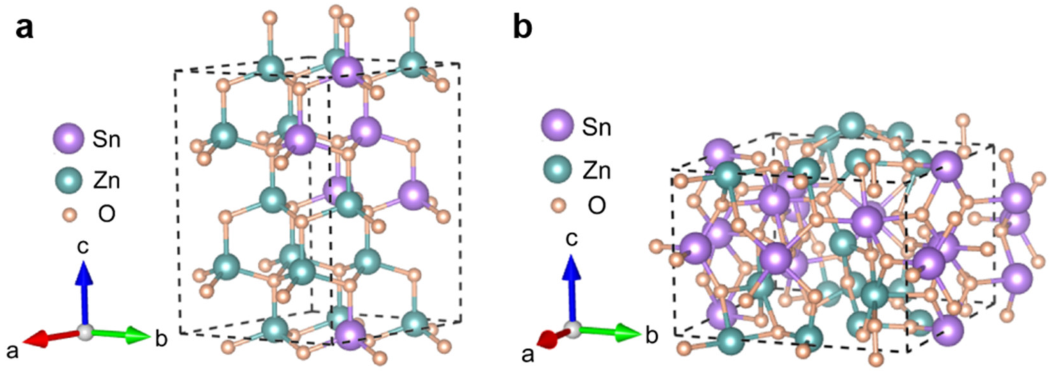

Supplementary Figure 13. The optimized structure of ZTO fibers. a ZTO-7:3 fibers. **b** ZTO-1:1 fibers.

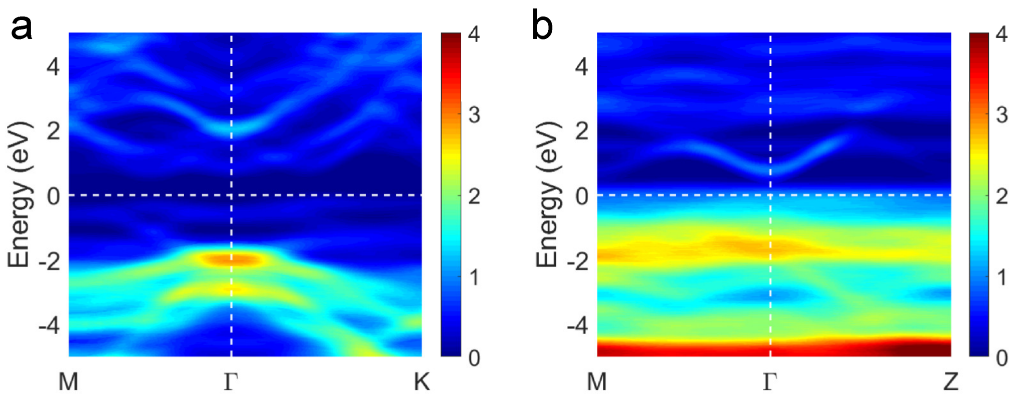

Supplementary Figure 14. Energy band structure of ZTO fibers. a ZTO-7:3 fibers. **b** ZTO-1:1 fibers.

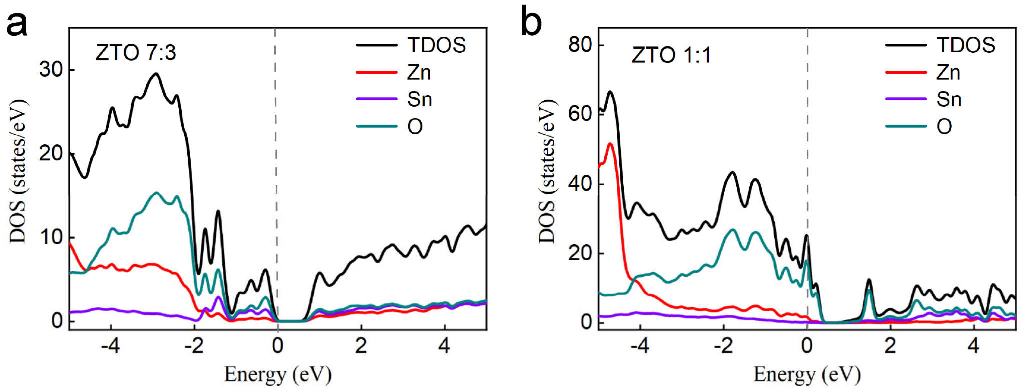

Supplementary Figure 15. DOS of ZTO fibers. a ZTO-7:3 fibers. **b** ZTO-1:1 fibers. The Fermi level is set to zero.

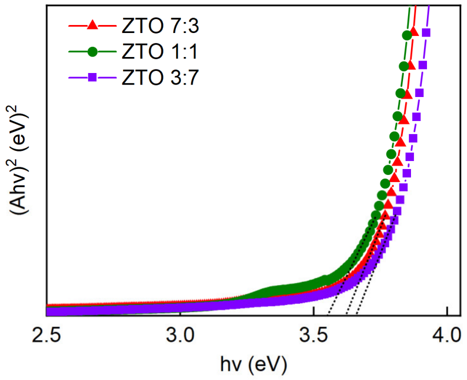

Supplementary Figure 16. Band gap measurement of ZTO fibers. Plot of $(Ahv)^2$ as a function of photon energy hv for three samples.

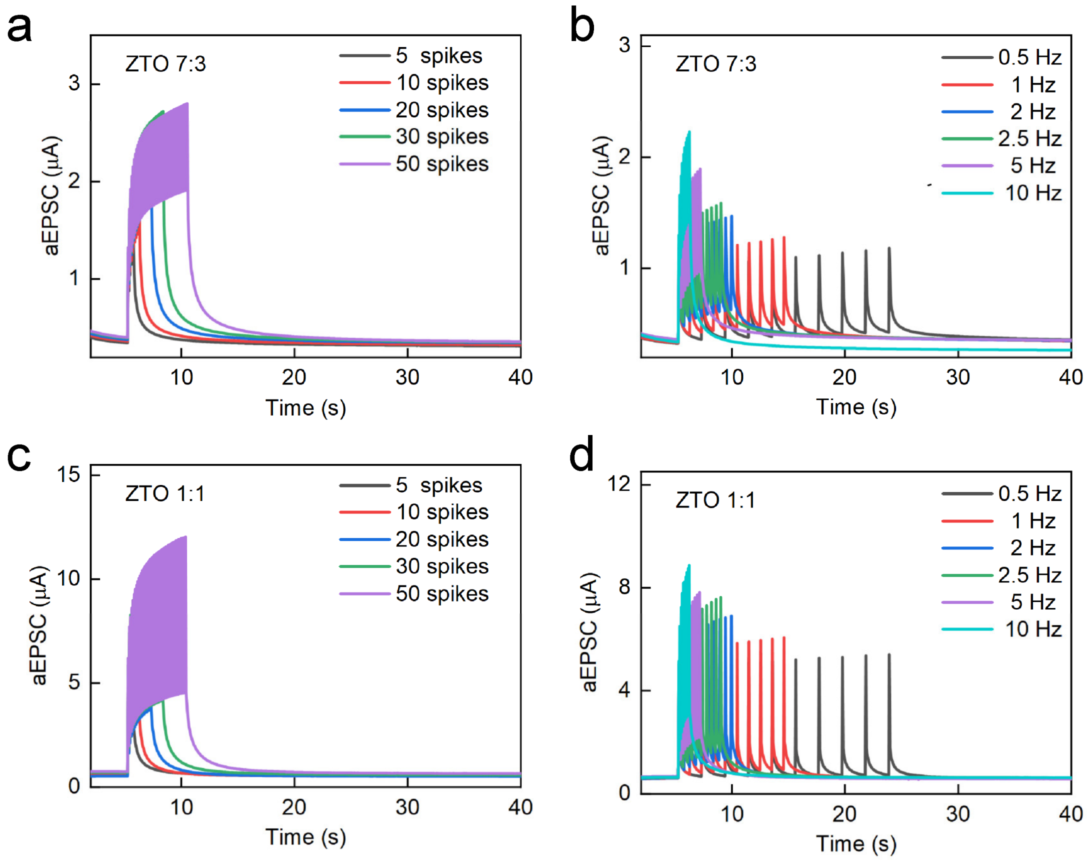

Supplementary Figure 17. aEPSC of ZTO artificial synapses triggered by consecutive spikes (2 V, 50 ms) with spike numbers 5, 10, 20, 30 or 50, and with spike frequencies 0.5, 1, 2, 2.5, 5 or 10 Hz. aEPSC triggered by consecutive spikes with different spike numbers. **a** ZTO-7:3 artificial synapse. **c** ZTO-1:1 artificial synapse. aEPSC triggered by consecutive spikes with different spike frequencies. **b** ZTO-7:3 artificial synapse. **d** ZTO-1:1 artificial synapse.

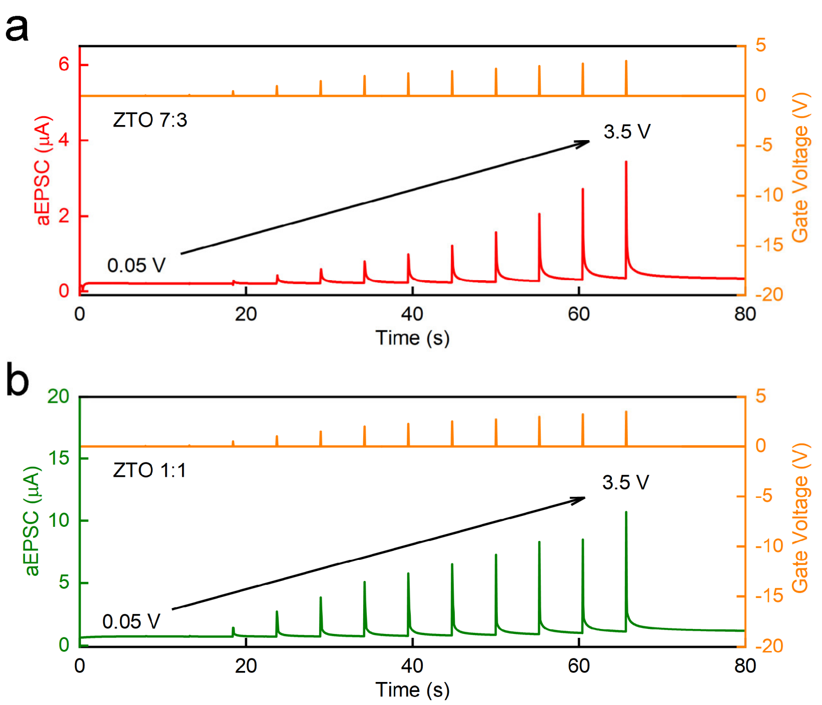

Supplementary Figure 18. aEPSC of ZTO artificial synapses triggered by spikes (50 ms) with amplitudes 0.05, 0.1, 0.5, 1, 1.5, 2, 2.25, 2.5, 2.75, 3, 3.25 or 3.5 V. a ZTO-7:3 artificial synapse. **b** ZTO-1:1 artificial synapse.

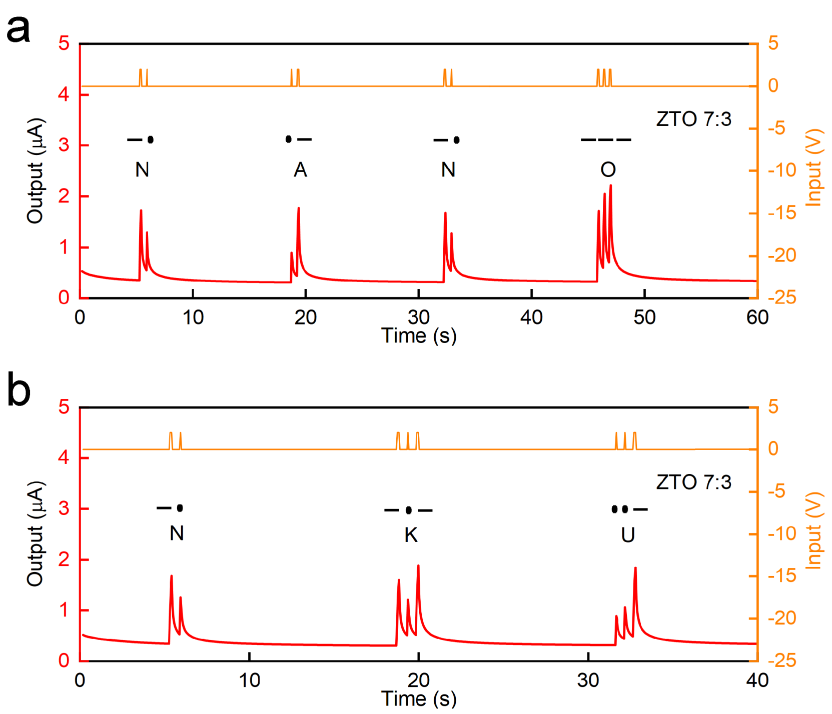

Supplementary Figure 19. International Morse code realized using ZTO-7:3 artificial synapse. a “NANO”. **b** “NKU”.

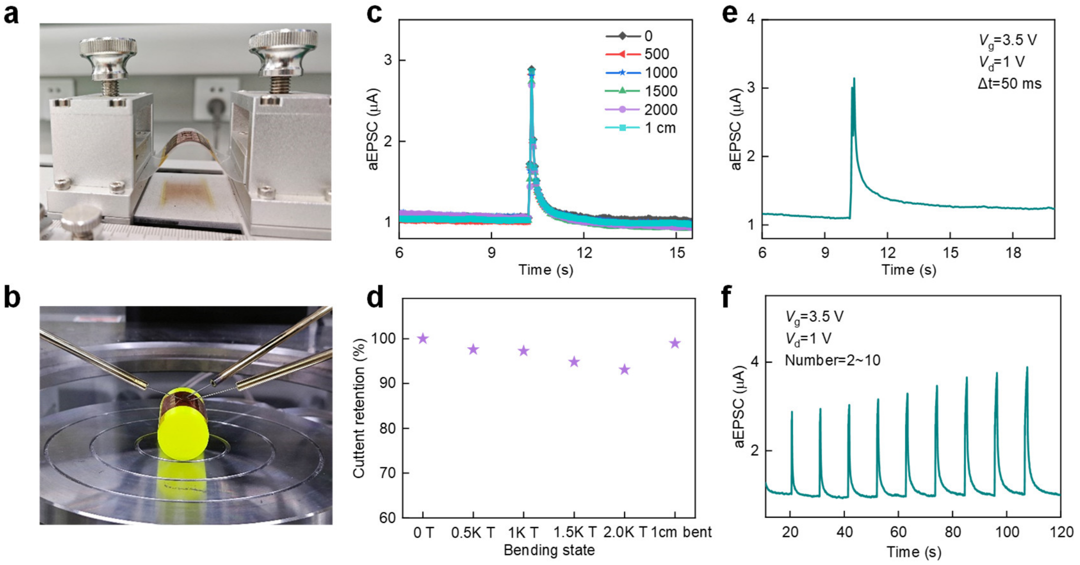

Supplementary Figure 20. Synaptic properties of the flexible ZTO-3:7 artificial synapse. a Digital image of the cyclic bending test of the device (bending 0, 500, 1000, 1500, and 2000 times). **b** Digital image of static bending test of the device (bending radius = 1 cm). **c** aEPSC of the flexible ZTO-3:7 artificial synapse under different bending states (0, 500, 1000, 1500, and 2000 bending times, bent at 1-cm radius). **d** Current retention (the ratio of aEPSC peak with/without bending) under different bending states. **e** aEPSC of the flexible ZTO-3:7 artificial synapse triggered by a pair of spikes (3.5 V, 50 ms) at Δt (time intervals between two spikes) = 50 ms. **f** aEPSC of the flexible ZTO-3:7 artificial synapse triggered by consecutive spikes (3.5 V, 50 ms) with spike numbers 2, 3, 4, 5, 6, 7, 8, 9, and 10.

Note: The fabrication process of the flexible artificial synapse is as follows:

A polyimide (PI) substrate was ultrasonically cleaned in deionized water, isopropanol, and anhydrous ethanol for 30 min, sequentially. Then digitally-aligned ZTO fibers (molar ratio of Zn:Sn = 3:7) were printed on the substrate surface to produce the channel. The printed ZTO fibers were annealed in a muffle furnace at 350 °C for 2 h. Gold source and drain electrodes (80 nm) were then thermally deposited through a shadow mask. Finally, the prepared PVDF-HFP/[EMIM-TFSI] ion gel was transferred onto the ZTO-fibers channel area.

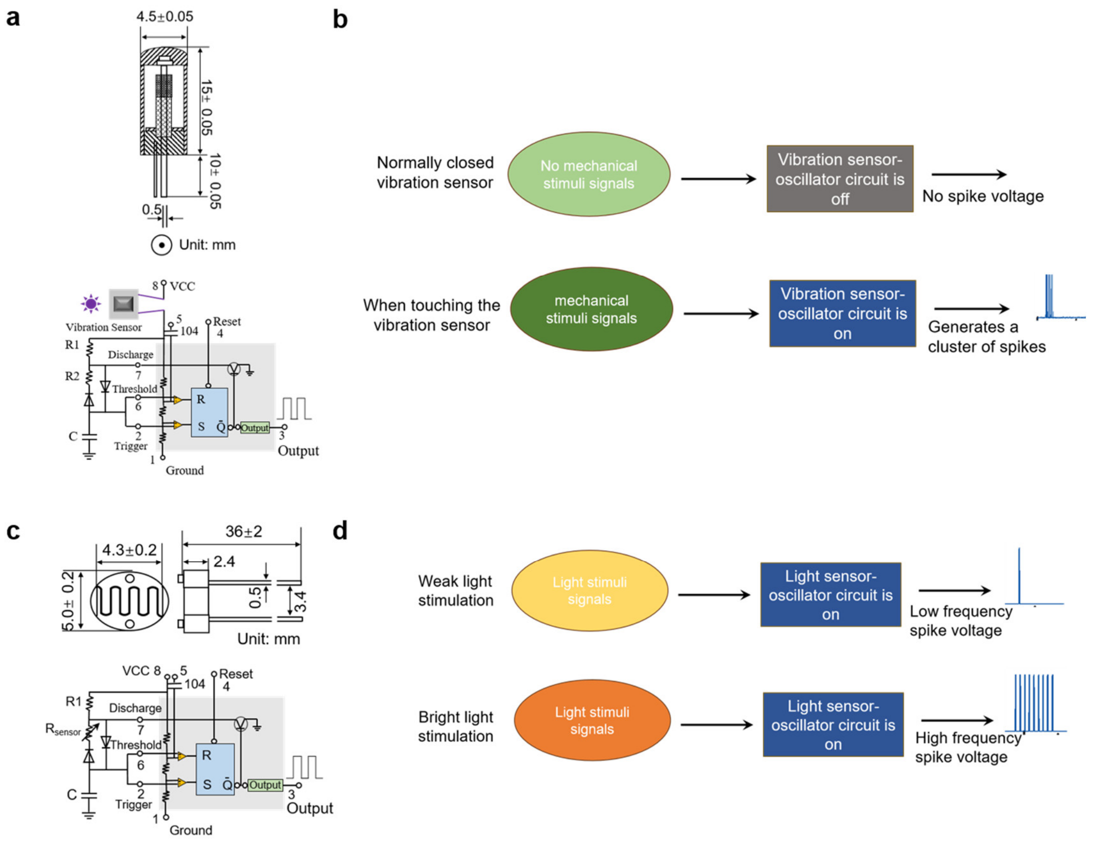

Supplementary Figure 21. Sensors and sensor-oscillation circuits. a Vibration sensor and corresponding sensor-oscillation circuit. **b** Schematic of operation and working mechanism of vibration sensor. **c** Light sensor and corresponding sensor-oscillation circuit. **d** Schematic of operation and working mechanism of light sensor. The sensor-oscillation circuit can encode stimulus information by spike frequency. The somatosensory and light neural communication by integrating ZTO artificial synapses with sensors (vibration-threshold or light-dependent resistor) by an improving sensor-oscillation circuit. A commercially-available vibration sensor (SW-180 10P) and photosensitive resistor (T5506) were used to ensure the operational stability of the sensor-oscillation circuit.

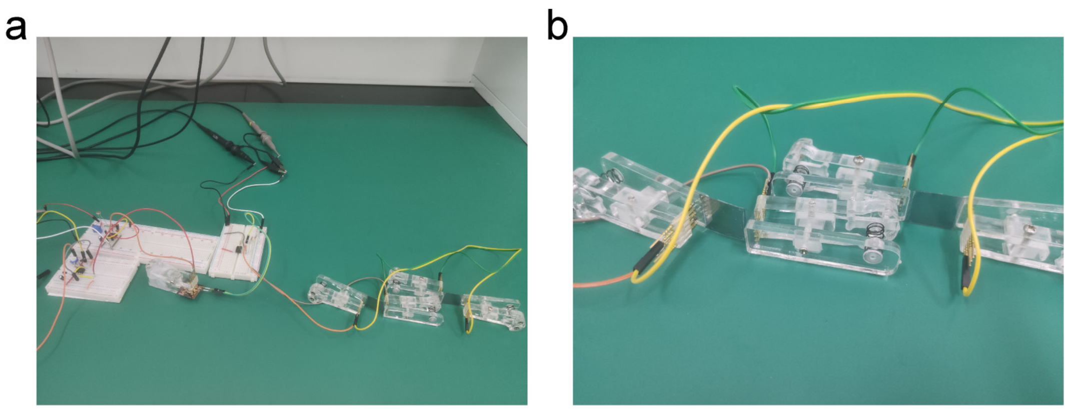

Supplementary Figure 22. Digital images of artificial corneal reflex arc and electrochromic actuators. a Artificial corneal reflex arc. **b** A pair of electrochromic actuators.

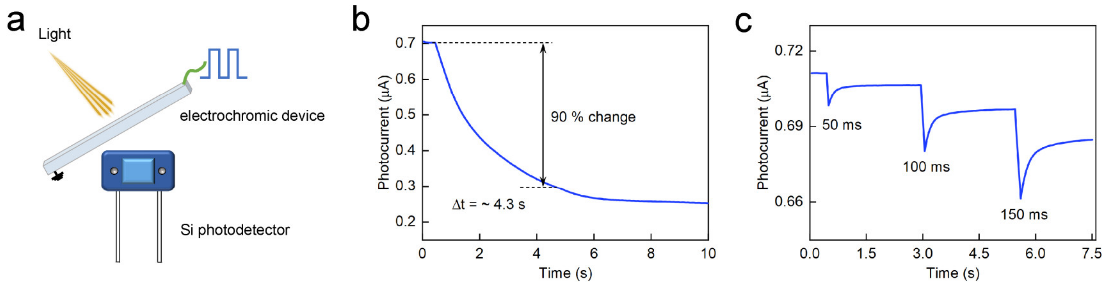

Supplementary Figure 23. Measurement of the temporal response information of the electrochromic device. a Schematic of the configuration in the test. **b** Time-dependent photocurrent during coloring of the electrochromic device. **c** Response of the electrochromic device under the applied voltages with different duration (50, 100, and 150 ms). The transmittance change of the electrochromic device was displayed by the change of photocurrent collected from the photodetector. The response time was extracted from time-dependent photocurrent during color change of the electrochromic device.

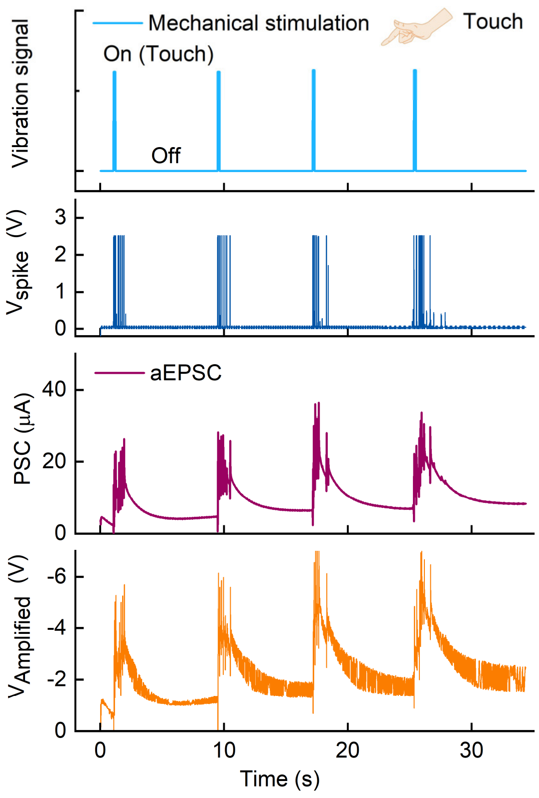

Supplementary Figure 24. Statistical curve of signal output over time for each part of the artificial corneal reflex arc under mechanical stimulation.

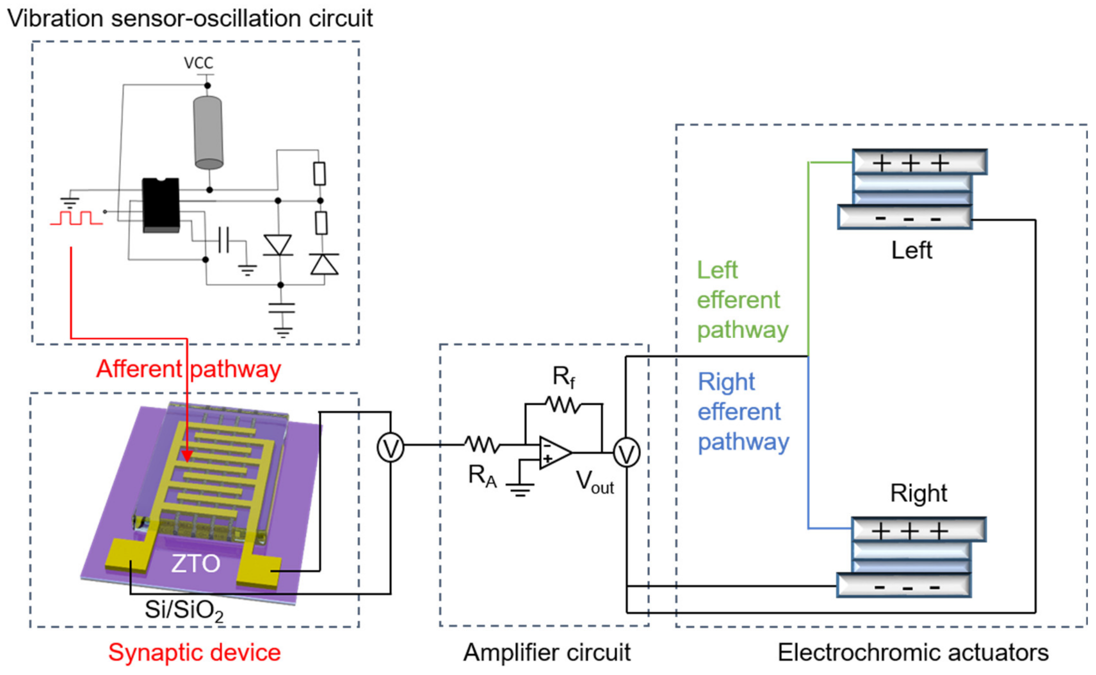

Supplementary Figure 25. Schematic of the artificial corneal reflex arc evoked by mechanical stimuli.

Supplementary Tables

Supplementary Table 1. Structural and chemical properties of ZTO fibers with different Zn:Sn molar ratios and their corresponding synaptic characteristics

Molar ratios (Zn:Sn)	Crystal structure	Area ratio of O_b peaks	aEPSC peak (2 V, 50 ms)	Synaptic plasticity
7:3	Wurtzite	0.18	0.83 μA	STP
1:1	Amorphous	0.30	4.61 μA	STP
3:7	Rutile	0.43	12.98 μA	LTP

Supplementary Table 2. Comparison of transmittance (T) and haze (H) between ZTO fibers and other materials

Material	Year	T (%)	H (%)	Morphology	μ_n	Toxicity
**ZTO (this work)**	**2023**	**99.89**	**0.36**	**fibers**	**12.8**	**No**
AgNW[S11]	2016	98.7	1	Nanowires	2.08	No
AgNW[S12]	2018	95	1.2	Nanowires	/	No
AgNW[S13]	2019	99.8	0.47	Nanowires	/	No
AgNW[S14]	2020	99.6	0.56	Nanowires	/	No
CuNW[S15]	2014	97	4	Nanowires	/	No
CuNW[S16]	2018	96.65	3.5	Nanowires	/	No
ITO[S17]	2021	90.61	0.08	Thin film	/	Yes
AZO[S18]	2015	95	1.8	Thin film	18.1	No
FTO[S19]	2019	96.38	5.11	Thin film	/	Yes
PEDOT:PSS[S20]	2017	90.1	0.1	Thin film	/	No
CNT[S21]	2015	90.8	1.6	Nanotubes	/	Yes
GO[S22]	2013	92	2.7	Nanotubes	/	Yes
Graphene[S23]	2016	94	0.5	Thin film	/	Yes

T: transmittance. H: haze. μ_n: carrier mobility [$cm^2/(V{\cdot}s)$]. NW: nanowires. CNT:

carbon nanotube. GO: graphene oxide.

Supplementary Table 3. **Band gap of ZTO fibers with different Zn:Sn molar ratios obtained by experiment and simulation**

Samples	Bandgap (eV) Experiment	Bandgap (eV) Simulation
ZTO 7:3	3.62	0.75
ZTO 1:1	3.54	0.53
ZTO 3:7	3.66	1.58

Supplementary Table 4. **The different states of electrochromic actuators under the same stimulus and the corresponding neural damage**

Stimulus	Left eye	Right eye	Reflex type	Damage
Right eye	Close	Close	Bilateral	Healthy
Right eye	Open	Open	/	Afferent pathway or central nervous system
Right eye	Open	Close	Ipsilateral	Contralateral efferent pathway
Right eye	Close	Open	Contralateral	Ipsilateral efferent pathway

Supplementary References

S1. Zunger, A., Wei, S., Ferreira, L. G. & Bernard, J. E. Special quasirandom structures. *Phys. Rev. Lett.* **65**, 353-356 (1990).

S2. Kresse, G. & Hafner, J. Ab initio molecular dynamics for open-shell transition metals. *Phys. Rev. B: Condens. Matter* **48**, 13115-13118 (1993).

S3. Kresse, G. & Furthmüller, J. Efficiency of ab-initio total energy calculations for metals and semiconductors using a plane-wave basis set. *Comp. Mater. Sci.* **6**, 15-50 (1996).

S4. Blochl, P. E. Projector augmented-wave method. *Phys. Rev. B* **50**, 17953-17979 (1994).

S5. Perdew, J. P., Burke, K. & Ernzerhof, M. Generalized gradient approximation made simple. *Phys. Rev. Lett.* **77**, 3865-3868 (1996).

S6. Wang, V., Xu, N., Liu, J.-C., Tang, G. & Geng, W.-T. VASPKIT: A user-friendly interface facilitating high-throughput computing and analysis using VASP code. *Comput. Phys. Commun.* **267**, 108033 (2021).

S7. Choi, H. H., Cho, K., Frisbie, C. D., Sirringhaus, H. & Podzorov, V. Critical assessment of charge mobility extraction in FETs. *Nat. Mater.* **17**, 2-7 (2017).

S8. Jiang, C. *et al.* Mammalian-brain-inspired neuromorphic motion-cognition nerve achieves cross-modal perceptual enhancement. *Nat. Commun.* **14**, 1344 (2023).

S9. Coulter, J. B. & Birnie, D. P. Assessing Tauc plot slope quantification: ZnO thin films as a model system. *Phys. Status Solidi B* **255**, 1700393 (2018).

S10. Mao, J.-Y. *et al.* Lead-free monocrystalline perovskite resistive switching device for temporal information processing. *Nano Energy* **71**, 104616 (2020).

S11. Lee, Y. *et al.* Versatile metal nanowiring platform for large-scale nano- and opto-electronic devices. *Adv. Mater.* **28**, 9109-9116 (2016).

S12. Niu, Z. *et al.* Synthesis of silver nanowires with reduced diameters using benzoin-derived radicals to make transparent conductors with high transparency and low haze. *Nano Lett.* **18**, 5329-5334 (2018).

S13. Chen, G. *et al.* Water-based purification of ultrathin silver nanowires toward transparent conductive films with a transmittance higher than 99%. *ACS Appl. Mater. Interfaces* **11**, 22648-22654 (2019).

S14. Xiao, D., Navik, R., Tan, H., Gai, Y. & Zhao, Y. Sorting purified silver nanowires using supercritical carbon dioxide elutriation coupled with membrane for fabricating flexible and transparent conductive electrodes. *J. Supercrit. Fluid.* **164**, 104915 (2020).

S15. Mayousse, C., Celle, C., Carella, A. & Simonato, J.-P. Synthesis and purification of long copper nanowires. application to high performance flexible transparent electrodes with and without PEDOT:PSS. *Nano Res.* **7**, 315-324 (2014).

S16. Zhang, Y., Guo, J., Xu, D., Sun, Y. & Yan, F. Synthesis of ultrathin semicircle-shaped copper nanowires in ethanol solution for low haze flexible transparent conductors. *Nano Res.* **11**, 3899-3910 (2018).

S17. Chiang, J.-L., Li, S.-W., Yadlapalli, B. K. & Wuu, D.-S. Deposition of high-

transmittance ITO thin films on polycarbonate substrates for capacitive-touch applications. *Vacuum* **186**, 110046 (2021).

S18. Nian, Q. *et al.* Highly transparent conductive electrode with ultra-low haze by grain boundary modification of aqueous solution fabricated alumina-doped zinc oxide nanocrystals. *APL Mater.* **3**, 062803 (2015).

S19. Wang, L. K., Chen, J. J., Yu, J. Y., Zhao, H. L. & Yang, J. K. Highly textured spray-deposited SnO2:F films with high haze for solar cells. *Vacuum* **169**, 108879 (2019).

S20. Gueye, M. N., Carella, A., Demadrille, R. & Simonato, J. P. All-polymeric flexible transparent heaters. *ACS Appl. Mater. Interfaces* **9**, 27250-27256 (2017).

S21. Han, H. J., Choi, Y. C. & Han, J. H. Preparation of transparent conducting films with improved haze characteristics using single-wall carbon nanotube-silver nanowire hybrid material. *Synth. Met.* **199**, 219-222 (2015).

S22. Moon, I. K. *et al.* 2D graphene oxide nanosheets as an adhesive over-coating layer for flexible transparent conductive electrodes. *Sci. Rep.* **3**, 1112 (2013).

S23. Park, J. Y. *et al.* PDMS-paraffin/graphene laminated films with electrothermally switchable haze. *Carbon* **96**, 805-811 (2016).
