# Supplementary material for: An artificially-intelligent cornea with tactile sensation enables sensory expansion and interaction
Source: Nat Commun. 2023 Nov 7;14:7181. doi: 10.1038/s41467-023-42240-3 (PMC10630301; doi:10.1038/s41467-023-42240-3)
Supplement: Supplementary file 3 — Description of Additional Supplementary Files [file 41467_2023_42240_MOESM3_ESM.docx]

**Description of Additional Supplementary Files**

File Name: Supplementary Movie 1

Description: Gradual changes of electrochromic actuators under the real-time touch.

File Name: Supplementary Movie 2

Description: States of electrochromic actuators under bilateral reflex, ipsilateral reflex and contralateral reflex.

File Name: Supplementary Movie 3

Description: Gradual changes of electrochromic actuators under different light input intensities.
